# A new perspective on prostate cancer treatment: the interplay between cellular senescence and treatment resistance

**DOI:** 10.3389/fimmu.2024.1395047

**Published:** 2024-04-17

**Authors:** Meng-Yao Xu, Zhi-Yu Xia, Jian-Xuan Sun, Chen-Qian Liu, Ye An, Jin-Zhou Xu, Si-Han Zhang, Xing-Yu Zhong, Na Zeng, Si-Yang Ma, Hao-Dong He, Shao-Gang Wang, Qi-Dong Xia

**Affiliations:** Department of Urology, Tongji Hospital, Tongji Medical College, Huazhong University of Science and Technology, Wuhan, China

**Keywords:** cellular senescence, drug resistance, treatment resistance, prostate cancer, SIPS

## Abstract

The emergence of resistance to prostate cancer (PCa) treatment, particularly to androgen deprivation therapy (ADT), has posed a significant challenge in the field of PCa management. Among the therapeutic options for PCa, radiotherapy, chemotherapy, and hormone therapy are commonly used modalities. However, these therapeutic approaches, while inducing apoptosis in tumor cells, may also trigger stress-induced premature senescence (SIPS). Cellular senescence, an entropy-driven transition from an ordered to a disordered state, ultimately leading to cell growth arrest, exhibits a dual role in PCa treatment. On one hand, senescent tumor cells may withdraw from the cell cycle, thereby reducing tumor growth rate and exerting a positive effect on treatment. On the other hand, senescent tumor cells may secrete a plethora of cytokines, growth factors and proteases that can affect neighboring tumor cells, thereby exerting a negative impact on treatment. This review explores how radiotherapy, chemotherapy, and hormone therapy trigger SIPS and the nuanced impact of senescent tumor cells on PCa treatment. Additionally, we aim to identify novel therapeutic strategies to overcome resistance in PCa treatment, thereby enhancing patient outcomes.

## 1 Introduction

Prostate cancer (PCa) is a significant health concern for men globally, and in the United States, PCa is the most common malignancy and the second leading cause of death among men (1), with over 1.2 million new cases diagnosed worldwide annually (2–4).

The European Association of Urology (EAU) suggests stratifying PCa based on risk factors, such as prostate-specific antigen (PSA) and Gleason score (GS), to assist medical professionals in the evidence-based management of different risk PCa and predict recurrence risk after definitive treatment (5). Among newly diagnosed cases of localized low-risk PCa, active surveillance (AS) or radical prostatectomy is advocated as the primary treatment options, while the remaining 15-20% of high-risk PCa patients necessitate a comprehensive treatment regimen comprising chemotherapy, radiation therapy, and hormone therapy (4, 6).

The treatment landscape for PCa has come a long way in recent decades, but the challenge of treatment resistance persists, particularly in managing intermediate- and high-risk patients. Treatment resistance, classified as either primary (intrinsic) or secondary (acquired), continues to hinder effective treatment (7, 8). Primary resistance occurs when the cancer does not respond to treatment at the outset, while secondary resistance represents the local or distant recurrence of malignancy, following an initial clinical response (9–11). Hormone therapy remains a cornerstone for managing advanced PCa, but after a median treatment time of 18~24 months, the majority of these patients progress to castration-resistant prostate cancer (CRPC) (12, 13), underscoring the limitations of current treatments including first-line anti-androgen therapies like enzalutamide, which are not immune to the emergence of primary or acquired drug resistance (12, 14). Chemotherapy, particularly with docetaxel as the first-line regimen, plays a critical role in treating high-risk PCa (15, 16), either as a standalone treatment or in combination with other agents like abiraterone and prednisone for metastatic CRPC or as adjunct therapy post-surgery (16, 17). Docetaxel used as chemotherapy mainly interferes with the microtube network essential for cell mitosis and interphase cell function, leading to cell cycle arrest (18). However, chemotherapy often initially shows the desired effect, but they are prone to develop resistance over time, leading to treatment failure. Similarly, radiation therapy, which targets tumor cells through direct DNA physical damage or indirect damage from active oxygen free radicals (19–21). Cancer cells can adapt to radiation-induced apoptosis through dynamic interactions and regulation of multiple survival factors, ultimately leading to treatment failure (17, 22).

Previously, it was believed that the emergence of treatment resistance in tumor cells was due to genomic instability (23). This instability arises from genetic alterations such as gene mutations, amplifications, deletions, or chromosome translocations. These changes can lead to target proteins mutating and unable to bind to drugs or activate downstream signaling molecules or alternative survival pathways. As a result, tumor cells evade treatment, continuing to survive and proliferate (24, 25). These genetic changes might originate from cancer stem cells (CSCs), contributing to primary (intrinsic) resistance, or develop during treatment, leading to secondary (acquired) resistance.

With the advancements in technologies that capture the magnitude and dynamics of both genetic and non-genetic intratumor heterogeneity in the 4D (spatial and temporal) space and at single-cell resolution (8), it is now understood that tumor cells may develop treatment resistance through non-genetic mechanisms. These on-genetic mechanisms mainly involve alterations in tumor cell phenotype, such as tumor cell plasticity (TCP) (9, 10), and adaptive changes in the tumor microenvironment (TME). TCP, through processes like epithelial-to-mesenchymal transition (EMT) (26, 27), transdifferentiation, and cancer stem cell formation (28), enables tumor cells to undergo irreversible phenotypic transitions. This versatility allows tumor cells to switch between proliferative, metastatic tumor cells, slow-cycling cells, and drug-tolerant persister (DTP) (29, 30). Currently, this adaptive non-genetic change is recognized as a common mechanism for treatment resistance in tumor cells.

Cellular senescence and the emergence of tumor treatment resistance are processes that transition from order to disorder. López-Otín et al. highlighted similarities between cellular senescence and cancer, identifying four meta-hallmarks of cellular senescence (genomic instability, epigenetic alteration, chronic inflammation, and dysbiosis) and four hallmarks of cancer (genomic instability & mutation, epigenetic reprogramming, tumor promoting inflammation, and polymorphic microbiomes) (31, 32). This similarity underscores the critical interaction between cellular senescence and tumor progression. In 1965, Hayflick’s discovery that normal human cells in culture have a limited capacity to divide, known as “Hayflick limit”, serves as a marker of cellular senescence, and the process is referred to as “replicative senescence” (33). Additionally, exposure to external stimuli such as reactive oxygen species (ROS) (34), DNA-damaging agents (35), or activated proto-oncogenes can induce stress-induced premature senescence (SIPS), resembling the senescent cell phenotype (36, 37). Similarly, local ionizing radiation inflicts DNA damage in PCa cells, both directly and indirectly through increasing ROS, akin to chemotherapy effects (38). High-dose chemoradiation triggers tumor cell apoptosis, while low-dose promotes SIPS in tumor cells. These senescent tumor cells secrete various chemotactic factors, cytokines, and extracellular matrix (ECM), which induces phenotypic transition (EMT, transdifferentiation, and CSCs formation) in other tumor cells, leading to treatment resistance (39).

This review aims to examine the current status of treatment resistance in PCa and explore its interaction with cellular senescence. By providing a comprehensive overview of treatment resistance in PCa, it assists clinician in better understanding and addressing this challenge in practice.

## 2 Cellular senescence

Cellular senescence is accompanied by changes in molecular markers, in addition to the degenerative changes in cellular morphological structures. Senescence-associated β-galactosidase (SA-β-Gal) activity increase is one of the important biomarkers of cellular senescence (40, 41). This enzyme hydrolyzes X-Gal substrate to produce insoluble blue crystals in cytoplasm (42), which can be observed under optical microscope. In senescent cells, lysosomes swell and increase, leading to a large accumulation of SA-β-Gal in lysosomes and a shift in pH from the normal range of 4.5 to 6.0 (43, 44). Another typical feature of senescent cells is the senescence-associated heterochromatin foci (SAHF), which are highly folded and tightly bound DNA double-strand structures that prevent DNA expression (45, 46). After DAPI staining, punctate heterochromatin structures can be observed in the cell nucleus under fluorescence microscope, indicating SAHF’s role in inhibiting the expression of genes regulated by transcription factors such as MCM3, PCNA, or cyclin A (47). Additionally, senescent cells develop a senescence-associated secretory phenotype (SASP) (48–50), consisting of a group of pro-inflammatory (IL-1, IL-6, TNF-α), pro-angiogenic (VEGF, FGF), and growth stimulating factors (amphiregulin, epiregulin, angiogenin) (51, 52). The NF-κB signaling pathway plays a key role in regulating SASP expression (52, 53), while other signaling pathways such as cGAS-STING, p38 MAPK, and mTOR are also closely related to SASP expression (49, 50, 54).

**Figure 1:**
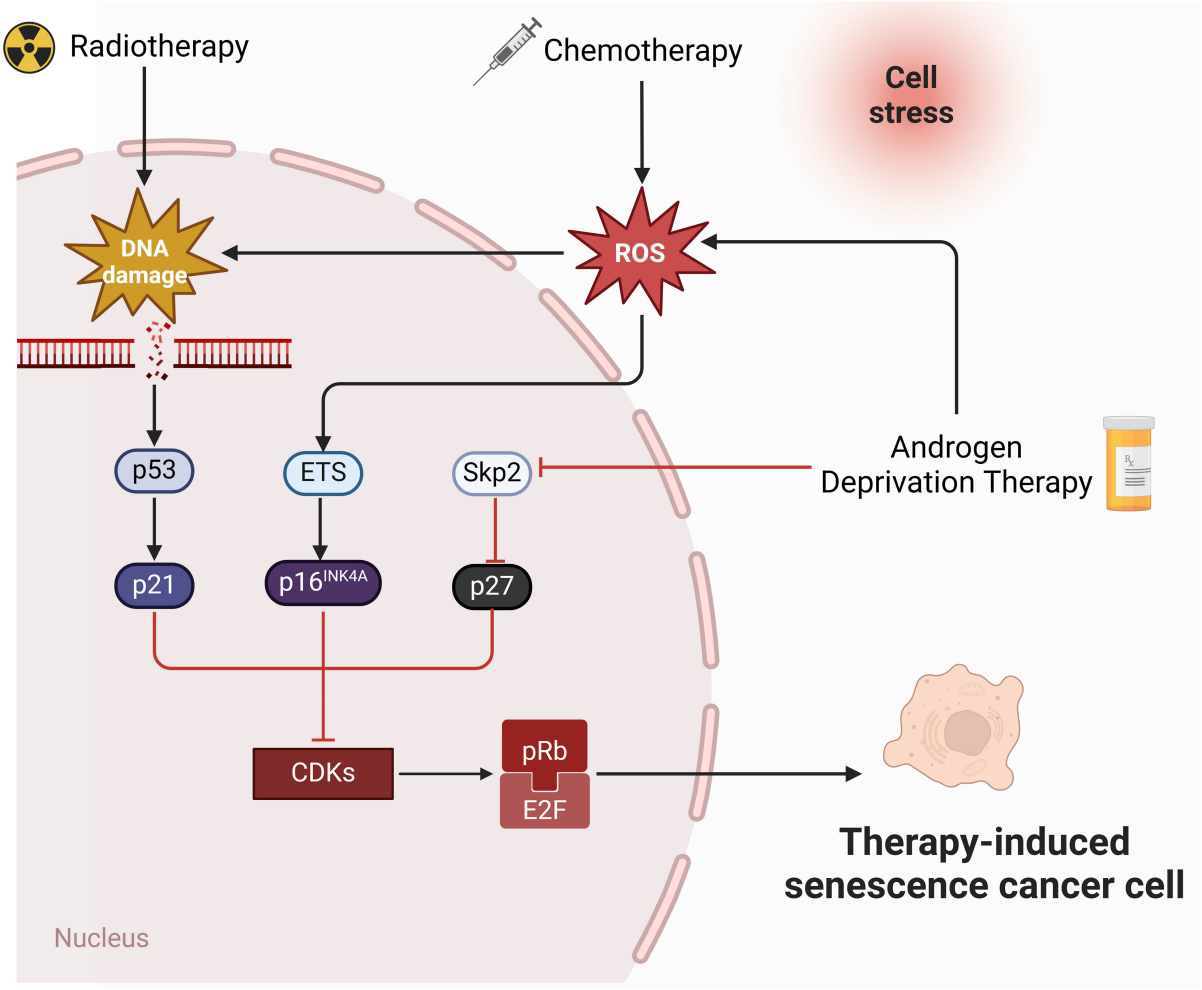
Molecular pathways of therapies-induced senescence in PCa. Radiotherapy and chemotherapy lead to irreversible DNA damage, ultimately triggering the activation of p53 and p21. p21 inhibits CDKs and mediates senescence by preventing the phosphorylation of Rb. In addition, chemotherapy induces senescence through ROS-ETS-p16 pathway. Androgen deprivation therapy induce senescence through Skp2-p27 pathway (created in Biorender.com).

The molecular mechanism of cellular senescence is a complex and multifaceted process that involves the interaction of multiple pathways and factors. The P53-P21 pathway is crucial for cellular senescence, primarily causing telomere shortening and cellular senescence caused by DNA damage (55). Upon DNA damage, P53 initiates the expression of its downstream gene, p21, which encodes a Cyclin-dependent protein kinase (Cdk) inhibitor to inhibit the Cyclin E-CDK2 activity. This prevention stops Cyclin-Cdk from phosphorylating Rb, keeping Rb unphosphorylated. The unphosphorylated Rb maintains binding with E2F, preventing the activation of the transcription regulator E2F, which ultimately results in cell cycle arrest at the G1 phase (56, 57). The p16-pRb pathway also matters for cellular senescence (58). p16 impedes the phosphorylation of the Cyclin D-CDK4/6-PBRB complex (59), keeping PRB in an active, non-phosphorylated state. This state allows pRb to attach to E2F proteins, obstructing the transcriptional activation of effectors of the cell cycle progression target proteins (57). The efficacy of these mechanisms relies on p16 keeping pRb active and non-phosphorylated. Furthermore, the significance of the PTEN-p27 pathway in cellular senescence is undeniable. PTEN, known for its tumor-suppressing properties, possesses both lipid and protein phosphatase activities that counteract the PI3K/AKT pathway (60). Mutation or deletion in PTEN prevent the dephosphorylation of Phosphatidylinositol-3,4,5-triphosphate (PIP3), leading to the activation of the PI3K/AKT signaling pathway (61, 62). p27kip1, a Cyclin-dependent kinase inhibitor, attaches to CDK2, blocking its kinase activity and halting cell cycle progression. p27 has a high expression level in the G1 phase and reduces to a minimum level in the S phase (63). PTEN upregulates p27 expression and decrease cyclin D1 activity to regulates the G1-S transition (**Figure 1**).

## 3 Resistance to androgen deprivation therapy (ADT) and cell senescence in PCa

Currently, the main treatment methods for PCa include surgery (radical prostatectomy and focal ablation therapy) (64), radiotherapy, chemotherapy, and endocrine therapy, among which endocrine therapy is the first-line treatment for advanced metastatic PCa (5). Exposure of PCa to therapies targeting AR, different anticancer compounds, and ionizing radiation can induce a senescent phenotype, termed therapy-induced senescence (TIS) (65). The shared mechanisms of senescence and PCa encompass disruptions in proteostasis, dysbiosis of the microbiota, persistent chronic inflammation, and widespread immunosenescence (66). TIS may benefit patients in the treatment of PCa (67). A reason for this is the activation of inflammatory cytokines targeting tumor cells, which enhances the innate immune response (68). Nonetheless, senescent cells could also facilitate tumor growth (69). SASP possesses tumor-promoting properties, such as chronic inflammation, angiogenesis, stemness, migration, and invasion (70). Therefore, TIS is a double-edged sword that can lead to reduced or enhanced tumor growth. Investigating its role in different treatments can help address resistance in PCa treatment and provide new clinical solutions.

### 3.1 Development of ADT resistance

Therapies that suppress the secretion of testicular androgens or the activity of androgens are collectively known as ADT, serving as the hormonal treatment for PCa (71). ADT encompasses both castration treatments and anti-androgen therapies. Castration therapies are further classified into surgical castration, which is the removal of both testicles through surgery, and medical castration, employing luteinizing hormone-releasing hormone (LHRH) analogs to inhibit the production and secretion of androgens. Anti-androgen therapies involve the use of steroidal or non-steroidal anti-androgens that competitively bind to androgen receptors (ARs), thus suppressing the action of androgens (72). As advanced PCa evolves from being androgen-dependent to androgen-independent, it transitions into castration-resistant CRPC, in which cancer progression can be driven by even low levels of androgens (73, 74). In early-stage CRPC, there’s still dependence on the androgen receptor (AR) signaling pathway, with drugs like steroidogenesis inhibitors (abiraterone) and AR antagonists (enzalutamide), which are AR pathway inhibitors (ARPIs), emerging as frontline treatments for CRPC (13). Under the continuous action of ARPIs, resistance quickly becomes a new challenge, with CRPC gradually evolving into a tumor that does not depend on the AR signaling pathway, resulting in neuroendocrine prostate cancer (NEPC) (75). The development of ADT resistance depends on adaptive changes and reactivation of the AR signaling pathway, including intratumoral production of androgens in PCa tissues, AR gene amplification (76), point mutations in AR (77), and constitutively active AR splice variants (78).

### 3.2 ADT can induce cellular senescence

While contributing to the development of resistance in PCa, ADT can also induce cell senescence. Studies *in vitro* indicate that various PCa cell lines, such as LNCaP and LAPC-4, exhibit senescent phenotypes after being cultured in charcoal-stripped serum (CSS), which is a medium that depletes androgens along with other steroids, thyroid, and vitamin D3 hormones (79). Signs of this cellular senescence are characterized by SA-β-gal positive staining, formation of heterochromatin foci (signaling epigenetic alterations), and an upregulation in SASP components expression, for instance, insulin-like growth factor-binding protein 3 (IGFBP3) and tissue plasminogen activator B (80). However, AR-negative and androgen-independent PCa cell line PC3 does not undergo cellular senescence under ADT, highlighting the crucial role of AR signaling in the proliferation arrest induced by ADT, with AR signals facilitating the transition of cells from G1 to S phase (81). The senescence induced by ADT is partly mediated by Skp2-dependent cyclin-dependent kinase inhibitor p27^Kip1^ (80). Some studies also suggest that CSS and bicalutamide induce p27^Kip1^-independent cell senescence, a process that may be executed through ROS-induced DNA damage and the p16^INK4a^ pathway (82). Additionally, the upregulation of the transcription factor CCAAT/Enhancer Binding Protein (C/EBP β) could also propel the aging response (83).

Clinical research shows that aging in PCa cells can be induced by non-steroidal AR antagonists such as bicalutamide, enzalutamide, darolutamide, and ataric acid treatments. Bicalutamide, a first-generation AR antagonist, triggers senescence in PCa cells by elevating the levels of CDK inhibitors p16^INK4a^ and p27^Kip1^ (84). Enzalutamide and darolutamide are second-generation AR antagonists that inhibit cell proliferation and induce PCa cell aging through a mechanism involving p16^INK4a^ induction (85, 86). Atraric acid, a natural AR antagonist, suppresses proliferation and induces senescence in PCa cells—both androgen-dependent (LNCaP) and castration-resistant (C4-2), as well as in PCa-like tumors from prostatectomies via the p16^INK4a^-pRb-E2F1 CyclinD1signaling pathway (87).

### 3.3 Cellular senescence benefits ADT therapy

Cellular senescence was initially described as a physiological suppression mechanism of tumor cells, as the development of cancer requires cell proliferation (88). Senescent cells cannot respond to mitotic signals nor re-enter the cell cycle, preventing damaged or stressed cells from dividing and forming tumors (89). There is evidence that TIS can yield beneficial effects for ADT, including the activation of the immune system and upregulation of inflammatory cytokines targeting tumor cells (90). Senescent cells secrete various chemokines, cytokines, and small molecules as components of SASP (91). A component of SASP, IL-1, is capable of inducing or amplifying Senescence-associated growth arrest and generating a pro-inflammatory environment, crucial in recruiting immune cells, thereby hindering cancer progression (92, 93). Moreover, lysosomal β-galactosidase (GLB1) is typically elevated in senescent cells (79), with a rise in GLB1 mRNA signifying better PCa outcomes (94). Studies indicate an increase in GLB1 protein levels in PCa patient samples within a month after initiating ADT (95), suggesting TIS aids ADT in PCa.

### 3.4 Cellular senescence promotes drug resistance in PCa

ADT-induced senescence can develop phenotypes favorable for cell survival, potentially evolving into clinically observed castration-resistant PCa through senescence evasion, cell-autonomous reprogramming, and the promotion of tumorigenic SASP, thereby countering the effectiveness of ADT (96).

Cellular senescence is not a stable state but rather a transitional phase, where, amidst complex epigenetic reprogramming, some PCa cells can regain the ability to proliferate after androgen deprivation ceases, known as TIS escape (97). Studies show that anti-androgen enzalutamide can trigger a reversible state akin to senescence, with no evidence of cell death or DNA damage (98).

During PCa progression, cellular senescence is tightly associated with telomere reduction (99, 100). Studies reveal that cancer cells typically undergo telomere shortening with anti-androgen treatment, likely due to treatment-induced stress and proliferation demands (101). The senescence induced by telomere shortening may drive CRPC development, allowing cancer cells to escape treatment suppression (102). Anti-androgen treatments can provoke DNA damage, with senescent cells’ reduced ability to repair DNA potentially diminishing treatment outcomes (79). Additionally, cellular senescence might activate DNA damage response pathways, prompting cancer cells to employ escape mechanisms for ongoing survival and proliferation (82).

Under the influence of ADT, various apoptotic regulatory factors become dysregulated, including the upregulation of BCL-2 (103), altering the activity of transcription factors during cell senescence, leading to transcriptomic-level control and gene expression reprogramming (97), a process previously identified as crucial for developing castration resistance (104). Following ADT, senescent PCa cells exhibit decreased sensitivity to various chemotherapies, including docetaxel (82, 83).

Cell senescence is also related to the regulation of inflammation and immune responses, with ADT indeed promoting the expression of SASP (105), which can alter the tissue microenvironment, with certain SASP paracrine components exhibiting tumor-promoting characteristics (106). IL-6 and IL-8, as components of SASP, can stimulate inflammation, epithelial-mesenchymal transition (EMT), and invasiveness (107), in addition to directly interacting with and activating AR (108, 109). Senescent fibroblasts and tumor cells can encourage the proliferation of nearby cells through paracrine activation of mechanisms, including the ERK1/2 signaling pathway, in both *in vitro* and *in vivo* settings (110, 111).

## 4 Resistance to chemotherapy and cellular senescence in PCa

### 4.1 Overview of chemotherapy resistance in PCa

Chemotherapy, a prevalent treatment for various malignancies, but PCa was once considered to be insensitive to chemotherapy. Since its introduction in 2004, docetaxel has become increasingly pivotal in treating metastatic castration-resistant PCa (112). Combining docetaxel with ADT and radiation therapy has been reported to enhance the recurrence-free survival in non-metastatic, locally advanced PCa (113, 114). While docetaxel remains a cornerstone for treating advanced stages of PCa, including castration-resistant variants, resistance to it markedly narrows treatment options (115). Chemotherapy resistance in PCa arises from various factors, including alterations in drug targets, epigenetic modifications, DNA repair mechanisms, cell death inhibition, and epithelial-mesenchymal transition (EMT) (116). Notably, research indicates that circARHGAP29 overexpression instigates docetaxel resistance and aerobic glycolysis within PCa cells (117). The cholinergic muscarinic M1 receptor (CHRM1) directly contributes to PCa cells’ resistance against docetaxel (118).

### 4.2 Chemotherapy triggers cellular senescence in PCa

Chemotherapy also leads to cellular senescence in cancer cells and TME components (119). It’s widely recognized that most cancer cells undergo growth arrest or death following chemotherapy (120). However, a minority of cancer cells enter prolonged growth arrest, exhibiting signs of cellular senescence (121). Studies indicate that DTX induces cellular senescence in the TC-1 and B16 tumor cell lines, marked by growth arrest, positive β-galactosidase staining, and elevated p21Waf1 (p21) expression (122). Clinical research on cancer survivors treated with chemotherapy shows increased levels of various cellular senescence markers post-treatment (123). Another clinical study analyzed paraffin-embedded tissue sections from PCa patients treated with neoadjuvant paclitaxel chemotherapy before radical prostatectomy, revealing specific detection of lipofuscin staining in the stroma of paclitaxel-treated patients (124).

Various chemotherapy drugs trigger cellular senescence through different mechanisms, including DNA damage, oxidative stress, and DNA methylation changes (125). Moreover, cellular senescence can be induced by impacting their metabolism and function. Research treating the TC-1 tumor cell line with DTX has revealed DNA double-strand breaks before or during mitosis, leading to ongoing activation of cell cycle checkpoints and the progression of subcellular cellular senescence (122). Oxidative damage to mitochondria from chemotherapy drugs leads to a reduction in cellular energy and functional shutdown, resulting in cellular senescence (126). During chemotherapy, alterations in DNA methylation and enzymes related to DNA methylation significantly impair cell function and mediate tumor cell cellular senescence by activating SASP, promoting a chronic inflammatory state (127).

### 4.3 The dual impact of chemotherapy-induced cellular senescence on PCa treatment

Chemotherapy drugs induce cancer cell senescence via DNA damage, cell cycle arrest, and apoptosis—a strategy that curtails cancer progression by reducing the proliferative capacity of senescent cells (128). The therapeutic effects of chemotherapy-induced cellular senescence extend to altering the TME (129). Signaling molecules from senescent cells can trigger inflammation, activate the immune system, and bolster the assault on cancer cells (130). Immune-mediated anticancer effects contribute to the elimination of residual cancer cells, lowering recurrence risk.

However, the cellular senescence triggered by chemotherapy drugs is closely linked to PCa resistance, with cancer cells deploying multiple strategies to bypass or diminish the adverse impacts of cellular senescence.

Enhancement of stem-like characteristics: German researchers discovered that cells enduring extreme chemotherapy environments transition into senescence, with these senescent cells sharing distinctive traits with resistant cells, including enhanced stem cell gene expression and heightened self-renewal capabilities, unbound by maturity constraints (131).

Activation of the p53 signaling pathway: The p53 gene, crucial for tumor suppression (132), activates in response to DNA damage, guiding cell repair or apoptosis (133). Nevertheless, research has identified p53 pathway anomalies in certain PCa cells, complicating their chemotherapy responses (134), thus facilitating their evasion of TIS and fostering resistance (135, 136).

Altered cell cycle regulation: Chemotherapy drugs may induce cell cycle arrest and modify the expression of cycle-regulating proteins, affording cells additional repair time for DNA damage and triggering senescence, culminating in treatment resistance (137, 138).

**Figure 2:**
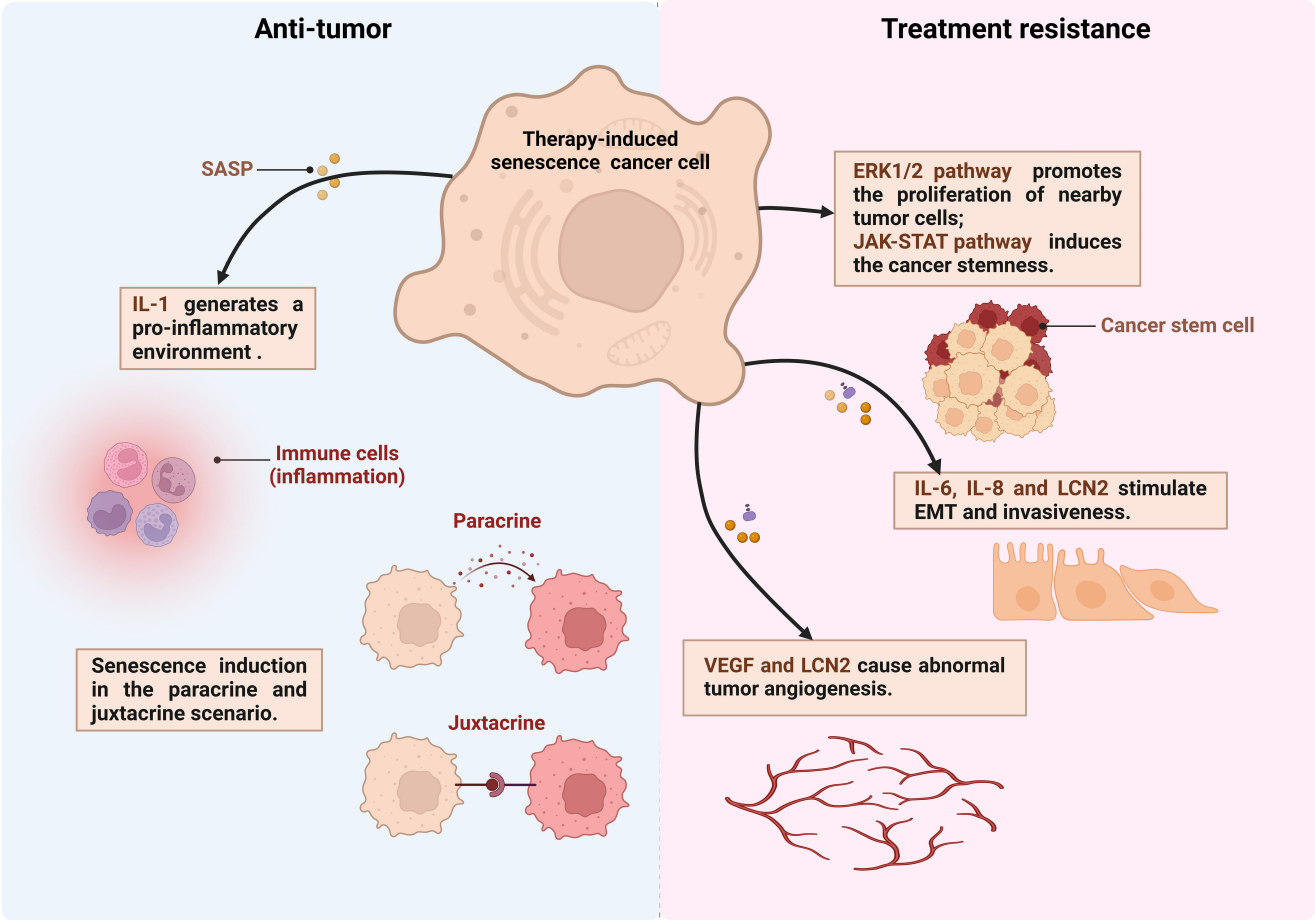
The dual effects of senescent cells in PCa. Therapy-induced senescent cancer cells secrete production of IL-6, IL-8, LCN2 and other senescence-associated secretory phenotype (SASP) factors. These factors exert juxtacrine and paracrine effects on the surrounding tumor microenvironment thereby anti-tumor or treatment resistance (created in Biorender.com). EMT, Epithelial-to-mesenchymal transition; VEGF, Vascular endothelial growth factor; LCN2, lipocalin 2.

Alterations of TME: Chemotherapy drugs foster a senescent phenotype in stromal fibroblasts, triggering metabolic shifts and the release of paracrine factors, activating tumor cell survival pathways such as ERK1/2 signaling pathways (139), and enhancing PCa cells invasiveness and clonogenic potential. These alterations may render the TME more supportive of resistance development (**Figure 2**) (124).

## 5 Cellular senescence and resistance to radiation therapy for PCa

Radiation therapy, recognized as the most widely used cytotoxic therapy, causes irreversible DNA damage, including double-strand breaks, single-strand breaks, DNA interstrand crosslinks, through γ rays or X-rays (140), This process initiates tumor cell apoptosis. However, the emergence of radiation therapy resistance poses significant challenges, particularly for patients with high-risk PCa (141–143).

Larsen et al. discovered that radiation-induced exogenous DNA damage activates Caspase-activated Dnase (CAD) (144), further triggering endogenous DNA breaks. This dual effect accelerates the aging of tumor cells through DNA damage-induced senescence (145). The resulting genomic instability activates the G2 cell cycle checkpoint, preventing tumor cells from entering the highest radiation sensitivity G2/M phase (146, 147), thereby acquiring radiotherapy resistance (144, 148). Additionally, senescent tumor cells secrete vascular endothelial growth factor (VEGF), leading to abnormal tumor angiogenesis and contributing to both chronic, diffuse hypoxia and acute, transient perfusion-related hypoxia. Pan-cancer cells often exhibit elevated levels of ANGPTL4, a protein that interacts with integrins to generate O2−, significantly contributing to tumor growth and survival (149). Additionally, ANGPTL4 is crucial in endothelial cells, regulating metabolism and angiogenesis. Specifically, endothelial-specific deletion of ANGPTL4 decreases pathological neovascularization and reduces permeability, emphasizing its crucial role in endothelial cell metabolism and angiogenic functions (150). Hence, ANGPTL4 represents a potential therapeutic target in the treatment of prostate cancer, it may be possible to disrupt tumor growth and survival mechanisms, offering a novel approach for the treatment of prostate cancer. In another study, Zhang et al. discovered that hypoxia-induced ANGPTL4 protein promotes radiation resistance in lung cancer through two mechanisms. Hypoxia not only increases the expression and secretion of ANGPTL4 in lung cancer cells, inhibiting ferroptosis and mediating radiation resistance, but also allows ANGPTL4 protein to be loaded into exosomes derived from hypoxic tumor cells. These exosomes then transfer to surrounding normoxic tumor cells, inducing radiation resistance by inhibiting ferroptosis through GPX4 (151). LCN2, a 25 kDa secreted glycoprotein belonging to the lipocalin family of lipid-carrying proteins, is highly expressed in PCa cells and can be induced by cellular senescence (152, 153). Studies have demonstrated that LCN2 interacts with MMP9 to form complexes, exerting pro-angiogenic and pro-tumor effects (154, 155). Furthermore, overexpression of LCN2 induces epithelial-mesenchymal transition in PCa, promoting tumor metastasis (156).

In addition, DNA damage-induced senescent tumor cells are closely linked to cancer stemness (157). Characterized by their tumorigenic nature, rapid multiplication, and multi-directional differentiation, CSCs play a pivotal role in determining tumor radio-sensitivity (158, 159). Tumor recurrence is also closely associated with CSCs, and these newly formed tumor cells, having been previously exposed to irradiation, exhibit reduced sensitivity to the initial radiation dose and gradually develop radiotherapy resistance. Evidence suggests that activating the JAK-STAT pathway can transform ordinary tumor cells into CSCs (160, 161). Studies have demonstrated that the JAK-STAT pathway is more highly activate in senescent than in non-senescent cancer cells (162). Meanwhile, certain signaling pathway that induce cellular senescence, such as the p53 and MAPK pathways, are known to activate the JAK-STAT pathway (163, 164). Furthermore, Karabicici et al. showed that the senescent tumor cells increase the mRNA expression of stem cell-related molecules, such as CD34 and CD133 (165).

**Figure 3:**
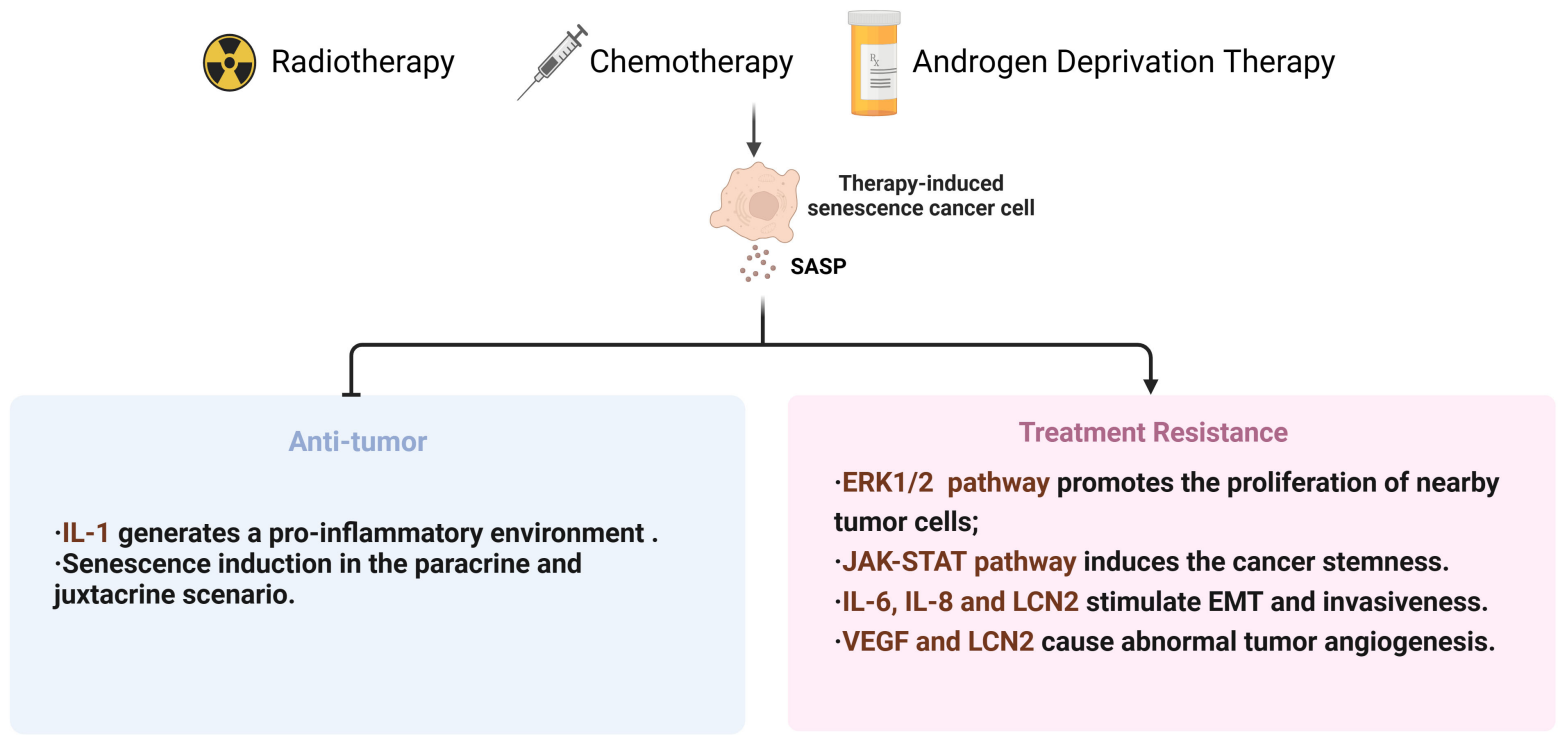
Summary diagram for therapies-induced senescence and dual effects in PCa. SASP, senescence-associated secretory phenotype; EMT, Epithelial-to-mesenchymal transition; VEGF, Vascular endothelial growth factor; LCN2, lipocalin 2.

For a long time, the radiotherapy resistance of PCa has been overlooked (166). With the increase of tumor resistance to radiation, it is necessary to gradually increase the radiation dose, which may be a dilemma for patients. On one hand, tumors can develop acquired resistance to radiation therapy after multiple exposures. On the other hand, bladder and rectal toxicities increase during radiation therapy. Hence, it is necessary to conduct research in two aspects: firstly, to explore strategies that can enhance the sensitivity of tumor radiotherapy, such as the application of nanoparticles (167) and the establishment of cancer radiosensitivity regulation factors database (dbCRSR) (168), to achieve effective local tumor control at acceptable and safe radiation dose; secondly, to investigate the mechanisms of tumor radiotherapy resistance, providing theoretical basis for improving the efficacy of radiotherapy (**Figure 3**).

## 6 The application and prospects of cellular senescence in PCa treatment resistance

### 6.1 The potential application of cellular senescence related markers in monitoring and prognostic evaluation of PCa therapy

As research into cellular senescence progresses, scientists have identified that biomarkers associated with cellular senescence offer promising applications in monitoring tumor treatment and assessing prognosis. In the context of PCa treatment, employing these biomarkers introduces innovative approaches for disease management and therapeutic intervention.

Biomarkers related to cellular senescence encompass an array of proteins, enzymes, and molecular signals, notably increased β-galactosidase activity, p16^INK4a, p21^CIP1/WAF1, and cyclin D1 (169). Significant changes in the expression levels of these markers during cellular senescence (170) make them valuable tools for monitoring and assessing the state of senescence.

Monitoring changes in senescence-associated biomarkers during PCa treatment offers a method to assess therapeutic effectiveness. Treatments like chemotherapy and radiation therapy can induce a senescent state in tumor cells, marked by a significant increase in the expression of senescence-related biomarkers (171). Regular monitoring of these biomarkers enables physicians to track treatment efficacy in real-time and promptly adjust therapeutic strategies as needed. Moreover, for innovative therapies that act by inducing tumor cell senescence, monitoring senescence-associated biomarkers directly indicates the efficacy of these drugs.

While biomarkers related to cellular senescence hold immense potential for therapy monitoring and prognostic evaluations, practical applications encounter significant challenges. Initially, cellular senescence, being a process influenced by numerous factors and stages, means a single biomarker might not adequately capture the complexity of the senescent state. Consequently, there is a need to devise methods for the combined detection of multiple biomarkers, aiming to boost the precision and dependability of monitoring efforts. Additionally, the expression patterns of senescence biomarkers may vary across different tumor types and among individuals, necessitating extensive validation and personalized assessment prior to their application.

### 6.2 Strategies for combating drug resistance in the treatment of PCa through cellular senescence

Cellular senescence, as a multifaceted biological phenomenon, has garnered significant interest in the realm of PCa treatment research in recent years. This process curtails tumor growth by inhibiting the proliferation of damaged or aberrant cells. In the context of tumor therapy, cellular senescence emerges as a potential double-edged sword. The accumulation of senescent cells can instigate inflammatory responses and alterations in the TME, potentially facilitating tumor progression. Tumor recurrence and severe, long-term adverse effects continue to pose significant challenges in the treatment of PCa patients. Consequently, the precise modulation of cellular senescence, harnessing its potential to combat treatment resistance in PCa, represents an emerging avenue of research (**Table 1**).

**Table 1:** Core literature summary table.

No.	Literature Reference	Research Objective	Key Findings
1	Kallenbach J. et al. (65)	This review aims to provide and analyze different mechanisms of therapy-induced senescence (TIS) in prostate cancer (PCa) and their effects on the tumor.	1. The most prevalent analyzed pathways in PCa as TIS are the p53/p21^WAF1/CIP1^, the p15^INK4B^/p16^INK4A^/pRb/E2F/Cyclin D, the ROS/ERK, p27^Kip1^/CDK/pRb, and the p27^Kip1^/Skp2/C/EBP β signaling. 2. TIS by radiation is mediated through p53. 3. ADT-induced senescence is partially mediated by the cyclin-dependent kinase inhibitor p27^Kip1^, which might depend on Skp2. 4. In chemotherapy and androgen deprivation therapy (ADT) the ROS-ERK-ETS-p16^INK4a^ and the p27^Kip1^-pRb pathways are activated to induce TIS. 5. Senescent cells activate the innate immune response, which target tumor cells and kill them. However senescent cells secrete also soluble inflammatory growth factors (SASP) and extracellular vesicles like exosomes, which change tumor microenvironment and might promote tumor growth.
2	Carpenter VJ. et al. (96)	This review aims to summarize the evidence that ADT promotes a senescent response in PCa and postulate mechanisms by which senescence may contribute to the development of castration-resistance.	1. ADT-induced senescence may support castration resistant prostate cancer (CRPC) development via escape from senescence, by cell autonomous-reprogramming, and by the formation of a pro-tumorigenic senescence-associated secretory phenotype (SASP). 2. Escape from ADT-induced senescence is permissive for the development of resistant tumor cell variants which possibly contribute to the clinically observed “castrate resistant” PCa. 3. Despite evidence that TIS may confer some advantageous outcomes, such as activation of the immune system, an overwhelming amount of literature has instead supported the notion that TIS ultimately has adverse and deleterious effects.
3	Wang L. et al. (69)	This review aims to discuss how senescence can be induced in cancer cells and describe the distinctive features of senescent cancer cells and how these changes in cellular physiology might be exploited for the selective eradication of these cells (senolysis).	1. Mechanistically, many chemotherapies cause DNA damage in cancer cells, which triggers senescence through ATM–CHK2 and ATR–CHK1 kinase-mediated activation of the interconnected p53–RB pathways. 2. Radiotherapy can induce an irreparable DNA damage response that activates ATM or ATR and p53–p21 pathway-mediated apoptosis and cellular senescence. 3. SASP cytokines, chemokines and other factors that modulate immune cells can either promote or inhibit senescent cell clearance.
4	Ewald JA. et al. (67)	This review examines the current status of TIS-regulated mechanisms, agents, and senescence biomarkers with the goal of encouraging further development of this approach to cancer therapy.	1. TIS can be induced in cancer cells lacking functional p53 and retinoblastoma protein using specific anticancer compounds or radiation. 2. TIS may lead to reduced toxicity-related side effects and increased tumor-specific immune activity. 3. Further development of TIS in cancer treatment could be facilitated by identifying additional compounds and targeted approaches for senescence induction.
5	Milanovic M. et al. (131)	This article aims to investigate whether chemotherapy-induced senescence could change stem-cell-related properties of malignant cells.	1. Key signaling components of the senescence machinery, such as p16^INK4a^, p21^CIP1^ and p53, as well as trimethylation of lysine 9 at histone H3 (H3K9me3), also operate as critical regulators of stem-cell functions (which are collectively termed ‘stemness’). 2. Cells released from senescence re-entered the cell cycle with strongly enhanced and Wnt-dependent clonogenic growth potential compared to virtually identical populations that had been equally exposed to chemotherapy but had never been senescent. 3. Senescence-associated stemness is an unexpected, cell-autonomous feature that exerts its detrimental, highly aggressive growth potential upon escape from cell-cycle blockade, and is enriched in relapse tumors.
6	Coppé JP. et al. (91)	This review aims to emphasize the potential effects of the SASP on cell behavior in the context of tumor progression.	1. senescent cells can have deleterious effects on the tissue microenvironment. The most significant of these effects is the acquisition of a SASP that turns senescent fibroblasts into proinflammatory cells that have the ability to promote tumor progression. 2. Senescence induced by irradiation in PCa patients is associated with a significantly increased re lease of exosome-like microvesicles. This novel secretory phenotype depends on the activation of p53. 3. The propensity of PCa patients to relapse after chemotherapy may be due to the accumulation of senescent tumor cells with inflammatory characteristics.

ADT, androgen deprivation therapy; CRPC, castration resistant prostate cancer; PCa, prostate cancer; SASP, senescence-associated secretory phenotype; TIS, therapy-induced senescence.

Strategy 1: Targeted induction of senescence in tumor cells. Specifically inducing senescence in tumor cells can effectively inhibit their proliferation, thus decelerating tumor growth (169, 172). The challenge lies in accurately targeting and inducing senescence in tumor cells while sparing normal cells from adverse effects. Research indicates that the NF-κB signaling pathway plays a crucial role in triggering SASP, with targeted intervention in this pathway effectively inducing tumor cell senescence (173). Additionally, activating the p53 pathway emerges as an effective strategy for promoting cellular senescence (174). Inhibiting the interaction between p53 and its negative regulator MDM4 can rejuvenate p53 activity in melanoma cells, enhancing tumor cell suppression and chemotherapy sensitivity (175). Thus, employing small-molecule drugs to activate p53 allows for the targeted induction of tumor cell senescence without harming normal cells. Despite the potential of activating the p53 pathway to promote cellular senescence in cancer therapy, current research faces multiple challenges. One possible explanation for the failure of MDM4 small-molecule inhibitors is that they may be too specific and unable to fully reactivate p53, as MDM2, which is structurally related to MDM4, can also bind and block the activity of p53. Therefore, a dual-targeting approach is considered crucial for success (176). Additionally, protein degraders offer a novel approach to reactivation of wild-type p53 by tagging and degrading target proteins through the combination of interest proteins and E3 ubiquitin ligases (177). Furthermore, mRNA-based therapies have demonstrated the potential to treat cancers with p53 mutations (178).

Strategy 2: Eliminating senescent cells. Inducing senescence in tumor cells can inhibit their proliferation, yet the accumulation of senescent cells might entail adverse outcomes, including the promotion of inflammation and alterations in the TME (179). Consequently, devising strategies to eliminate senescent cells represents a critical approach to surmounting treatment resistance. “Senolytics” refers to a category of drugs designed to specifically target and eliminate senescent cells (180). By targeting survival signaling pathways unique to senescent cells, such as navitoclax which targets the Bcl-2 protein family, these drugs mitigate the influence of senescent cells on the surrounding microenvironment (181), thus diminishing the risk of tumor progression. Targeting senescent cells shows promise for disease alleviation, yet its clinical translation remains intricate. The nonspecific nature of senescent cells in both tumor and healthy tissues poses a challenge for “Senolytics” agents, requiring discrimination to minimize collateral damage. Heterogeneity of the senescence phenotype complicates universal agent development, necessitating personalized approaches. Long-term impacts and safety profiles of “Senolytics” therapy remain uncertain, necessitating rigorous preclinical and clinical testing.

Strategy 3: Modulating the TME. Besides acting directly on tumor cells, modulating the TME is also an effective strategy for overcoming treatment resistance in PCa (182–184). Through the secretion of SASP, senescent cells influence neighboring cells and tissues, thereby fostering alterations in the TME (185). Inhibiting SASP secretion or blocking its effects can mitigate the adverse influence of senescent cells on the TME, thus diminishing tumor cell resistance (186). Utilizing anti-inflammatory drugs to curb inflammatory responses, or antibodies to obstruct critical SASP factors like IL-6 and IL-8, can significantly enhance the TME and curb tumor growth (187). However, current challenges lie in identifying specific targets within the heterogeneous TME, developing agents with high selectivity and low toxicity, and understanding the long-term impact of modulating the TME on PCa progression and patient outcomes.

## 7 Conclusion

In conclusion, cellular senescence in PCa therapy reveals both significant potential and formidable challenges. A profound comprehension of the interplay between cellular senescence and treatment resistance in PCa not only aids in uncovering mechanisms of resistance but also opens avenues for devising novel therapeutic strategies. In the future, research into cellular senescence will be pivotal in formulating innovative approaches to circumvent or reverse resistance and enhance therapeutic efficacy. Strategies focused on eliminating senescent cells or adjusting their secretions promise to yield more efficacious treatment alternatives for PCa patients.

## Author contributions

M-YX: Writing – original draft, Writing – review & editing. Z-YX: Visualization, Writing – original draft. J-XS: Writing – review & editing. C-QL: Writing – review & editing. YA: Writing – review & editing. J-ZX: Writing – review & editing. S-HZ: Writing – review & editing. X-YZ: Writing – review & editing. NZ: Writing – review & editing. S-YM: Writing – review & editing. H-DH: Writing – review & editing. S-GW: Writing – review & editing. Q-DX: Writing – original draft, Writing – review & editing.
